# Solid-State Fermentation of *Ganoderma lucidum* on Brewer’s Spent Grain and Sawdust for Mycelium-Based Biomaterial Production

**DOI:** 10.3390/jof12100788

**Published:** 2026-10-20

**Authors:** Nelson Dueñas, Richelly Celedon, Doménica Salomé Araque Buitrón, María Alejandra Cruz

**Affiliations:** 1 Mycomaker, Av. Portugal y Catalina Aldáz, Quito 170135, Ecuador; mycomaker@gmail.com; 2 Ingeniería en Biotecnología, Facultad de Ingeniería y Ciencias Aplicadas, Universidad de las Américas, Quito 170125, Ecuador; richellyco@live.com (R.C.); araquedome@gmail.com (D.S.A.B.)

**Keywords:** *Ganoderma lucidum*, brewer’s spent grain (BSG), sawdust, solid-state fermentation, mycelium-based biomaterials, agro-industrial residues, compressive strength

## Abstract

*Ganoderma lucidum* is a widely studied fungal species due to its potential for producing mycelium-based biomaterials. In mycotecture applications, fungal mycelium colonizes organic substrates, forming composite structures that can be used as sustainable biomaterials. Meanwhile, the management of agro-industrial and wood residues remains an environmental challenge, as these materials are often discarded or burned. This study aimed to evaluate the effects of substrate composition and inoculum load on the recovered dry fraction obtained during the solid-state fermentation (SSF) of *G. lucidum* using brewer’s spent grain (BSG), an agro-industrial by-product of beer production, and sawdust, a wood-processing residue. An exploratory approach based on a reduced central composite design (CCD) was used to evaluate the effects of inoculum load and substrate composition on the recovered dry fraction. The apparent specific growth rate (µ) during the observed apparent exponential growth interval (days 3–6) and the corresponding doubling time (td) were also estimated. The treatment associated with the highest recovered dry fraction contained 40.13% (*w*/*w*) sawdust and 59.87% (*w*/*w*) BSG, with an inoculum load of 2.69% (*v*/*w*). Growth kinetics were subsequently estimated under these conditions. The apparent specific growth rate over days 3–6 was 0.6346 day^−1^, corresponding to a doubling time of 1.0922 days. Substrate composition was the main factor associated with variation in the recovered dry fraction. These findings suggest that BSG and sawdust may be suitable substrate components for valorization through the SSF of *G. lucidum*. However, the recovered dry fraction is an operational measurement and should not be interpreted as a direct quantification of pure fungal biomass. The resulting specimens produced in this study exhibited a density of 0.24–0.27 g cm^−3^ and a compressive strength of 550–700 kPa.

## 1. Introduction

The development of fungal-based biomaterials is commonly referred to as mycotecture [1], in which fungal mycelium grows on organic substrates and forms materials with stable three-dimensional structures [2]. These substrates are commonly composed of agricultural and forestry residues, such as rice husks, wheat straw, barley residues, sawdust, coffee residues, cacao husks, and other lignocellulosic materials, which provide suitable conditions for fungal growth [3].

This process is commonly performed through solid-state fermentation (SSF), which involves the growth of microorganisms on moist solid substrates with limited free water. The mycelium can be inoculated using liquid cultures, agar cultures, or colonized grains, followed by incubation under controlled environmental conditions [4]. Fungal growth depends strongly on factors such as relative humidity and temperature; during incubation, a relative humidity of approximately 98% and temperatures close to 25 °C are generally recommended to prevent substrate drying and promote mycelial development [5,6]. The solid substrate provides physical support for fungal growth while also serving as a source of nutrients required for mycelial development. Compared with liquid-state fermentation (LSF), SSF offers advantages for the valorization of agricultural and forestry residues, as it generally requires lower amounts of water and can reduce energy and equipment requirements, facilitating the development of cost-effective biotechnological processes [7,8]. These characteristics also make SSF particularly suitable for integration into circular-economy strategies through the conversion of low-value residues into value-added products. Cultivation on natural solid substrates provides the advantage of supplying multiple essential nutrients for fungal growth, thereby supporting its development. Additionally, economic analyses have shown that the total capital investment required for submerged fermentation may be up to 78% higher than that required for SSF, highlighting the economic advantages of solid-state processes [9]. Once the substrate is fully colonized, the mycelium-based material can be molded and subsequently dried or heat-treated to stop growth and stabilize the final biomaterial [10]. Despite these advantages, SSF also presents important challenges. The heterogeneous nature of solid substrates makes process control more complex than in submerged fermentation, particularly regarding heat and mass transfer, moisture distribution, aeration, and biomass quantification. These limitations may affect process reproducibility and represent important bottlenecks for scale-up and industrial implementation [11].

Fungal biomaterials have attracted increasing attention as sustainable alternatives to conventional materials such as plastics, leather, and polystyrene, due to their comparable properties and lower environmental impact [1]. However, despite the rapid development of this field in recent years, standardized substrate formulations for their production remain limited.

Several lignocellulosic and agro-industrial residues have been investigated as substrates for the production of mycelium-based biomaterials, including wheat straw, hemp hurds, rice husks, cottonseed hulls, sawdust, spent coffee grounds, orange peels, banana leaves, and brewer’s spent grain, either individually or combined with other agricultural residues. These studies demonstrate that substrate composition strongly influences fungal colonization and the physical and mechanical properties of the resulting biomaterials [12].

However, studies evaluating the combined use of brewer’s spent grain and sawdust for the cultivation of *Ganoderma lucidum* and the development of mycelium-based biomaterials remain limited. The present study explores this substrate combination by integrating the high nutrient content of brewer’s spent grain with the structural support provided by sawdust, contributing to the valorization of two abundant agro-industrial residues within a circular bioeconomy framework [13].

Several fungal species have been explored for this purpose, including *Ganoderma lucidum*, *Pleurotus ostreatus*, *Trametes versicolor*, *Fomes fomentarius*, *Schizophyllum commune*, *Irpex lacteus*, and *Hypholoma capnoides* [14,15]. Among these species, *G. lucidum* was selected for this study due to its strong ability to degrade lignin and its well-documented potential in biomaterial production. This fungus produces enzymes such as laccases and peroxidases, which facilitate nutrient assimilation and promote strong mycelial adhesion to the substrate [16,17]. These characteristics make it a suitable candidate for the development of mycelium-based materials. In addition, its use has been explored by companies such as Ecovative, MycoWorks, Mogu, and Mycomaker [15,18,19].

In addition, several studies have shown that biomaterials produced using *Ganoderma lucidum* can show compressive strength values comparable to those of certain synthetic materials. Compressive strength refers to the ability of a material to withstand applied loads without structural failure and is commonly expressed in units such as kg cm^−2^ or kPa [20]. This parameter is particularly important when evaluating the potential application of mycelium-based materials in construction and other structural contexts, as it relates directly to material integrity and safety [20]. An earlier study proved that biomaterials produced with *G. lucidum* exhibited higher compressive strength than materials produced using other fungal species such as *Irpex lacteus* and *Pleurotus ostreatus* [17].

For comparison, expanded polystyrene (EPS), a widely used synthetic material, presents compressive strength values of approximately 690 kPa [21], whereas biomaterials produced with *Ganoderma* spp. present compressive strength values in a range of 44.17–750 kPa, depending on the substrate used for the solid-state fermentation [17,22].

The formulation of the substrate plays a critical role in fungal growth and directly influences the density and physical–mechanical properties of the resulting biomaterial. To produce mycelium-based materials, a suitable solid culture medium is therefore needed. In this context, the present study aimed to evaluate a substrate formulation for the growth of *Ganoderma lucidum* using sawdust and brewer’s spent grain (BSG) residues.

The use of agro-industrial and forestry residues as substrates aligns with the principles of the circular economy, as it promotes the valorization of materials that would otherwise be discarded [23]. In this context, upcycling involves transforming these residues into value-added products, reducing environmental impacts while potentially lowering production costs through the efficient use of available resources [24]. Furthermore, solid-state fermentation (SSF) enables the sustainable use of such waste materials and contributes to improving the composition of the final product [23].

In Ecuador, the management of agricultural and wood residues has become an important environmental concern. These residues are often burned or disposed of in landfills, contributing to air pollution through the emission of carbon monoxide, carbon dioxide, and particulate matter. Ecuador has significant forestry resources, and approximately 1500 tons of wood residues are generated annually in the city of Guayaquil alone without adequate treatment [24], generating a considerable environmental impact. Another residue produced in enormous quantities in Ecuador is BSG, with an estimated annual production of approximately 120,000 tons. When not effectively managed, this byproduct decomposes and releases greenhouse gases [25].

Both brewer’s spent grain (BSG) and sawdust are lignocellulosic residues with potential as components of substrates for fungal cultivation; however, their complementary chemical compositions make their combination particularly suitable for mycelium-based biomaterial production. BSG is a nutrient-rich byproduct containing fiber, carbohydrates, and proteins, with reported protein contents generally ranging from approximately 10 to 26% on a dry-weight basis [23,26,27]. Its relatively high protein content makes BSG a valuable source of nitrogen and other nutrients for fungal growth [28]. In contrast, sawdust is predominantly composed of lignocellulosic components and can provide a carbon-rich structural matrix that supports fungal colonization and contributes to the physical properties of the resulting biomaterial [29,30]. Therefore, combining BSG and sawdust can provide complementary nutritional and structural functions, while the fibrous nature of both residues contributes to the physical support and integrity of the mycelium-based material.

Therefore, this study aimed to evaluate the effects of substrate composition and inoculum load on the recovered dry fraction of *Ganoderma lucidum* during solid-state fermentation using sawdust and brewer’s spent grain (BSG) as substrates. A central composite design (CCD) was used to determine the influence of these factors and identify the conditions associated with the highest recovered dry fraction. Subsequently, the model-identified conditions were used to estimate the growth kinetics of *G. lucidum*, including the specific growth rate and doubling time. The results were further used to support the development of mycelium-based biomaterials from these agro-industrial and forestry residues.

## 2. Materials and Methods

### 2.1. Raw Material Reception and Pre-Treatment

BSG and sawdust were used as substrates for fungal cultivation. The sawdust used in this study was Pigüe (*Piptocoma discolor*), with an approximate particle size of 0.8–4.0 mm, and was obtained from the province of Pastaza, Ecuador. In addition, the brewer’s spent grain (BSG) was sourced from Andes Brewing Co., located in Quito, Ecuador. Both residues were received and dried on aluminum trays in a laboratory oven (BIOBASE, model BOV-V230F, Jinan, China) at 80 °C for approximately 24 h to reduce their initial moisture content and obtain more controlled and reproducible conditions for the experiments. The total weight of each residue was recorded before and after drying, and the moisture content was calculated based on the weight loss during the drying process. Drying was conducted until a constant weight was reached. Under these conditions, the final moisture content was approximately 9.3% for BSG and 2.9% for sawdust, ensuring that both materials were sufficiently dry, stable, and suitable for storage and later use in the experiments.

### 2.2. Raw Material Characterization

All raw-material characterization analyses (ash, nitrogen, and protein content) were performed in triplicate, and the results are reported as the mean of three independent replicates.

#### 2.2.1. Ash Content

Ash content was found using the ash method [31]. Briefly, 2 g of each dried sample was placed in crucibles and incinerated at 550 °C for 2 h. The ash percentage was calculated using Equation (1).

(1) $$\mathrm{Ash}\left(\%\right)\;=\frac{100\times \left(\mathrm{crucible weight with dry sample}-\mathrm{dry empty crucible weight}\right)}{\left(100-\%\mathrm{moisture}\right)\left(\mathrm{sample weight}\right)}$$

#### 2.2.2. Nitrogen and Protein Content

Total nitrogen content was quantified using the Kjeldahl method [32]. For each sample, 1 g of substrate was digested with sulfuric acid (12.5 mL for BSG and 10 mL for sawdust). Nitrogen content was calculated using Equation (2), and protein content was estimated using Equation (3), with a conversion factor (F) of 6.25 [33].

(2) $$\mathrm{Nitrogen}\;(\%)\;=\frac{\mathrm{ml titrant acid}\cdot \mathrm{acid Normality}\cdot 1.4007}{\mathrm{sample weight in grams}}$$

*Protein* (%) = %*N* × (*F*) (3)

### 2.3. Fungal Strain and Maintenance

The *Ganoderma lucidum* strain used in this study was obtained commercially from Fungi Andino. The supplier did not provide a specific strain name or identification code. The strain was cultivated on Potato Dextrose Agar (PDA; Merck KGaA, Darmstadt, Germany) plates and inoculated using previously colonized mycelial fragments. Cultures were incubated at 25 °C for 10 days until the Petri dishes were fully colonized [34].

### 2.4. Liquid Culture Medium Preparation and Incubation

The liquid culture medium consisted of 40 g L^−1^ glucose (Merck KGaA, Darmstadt, Germany) and 12 g L^−1^ yeast extract (Merck KGaA, Darmstadt, Germany), adjusted to pH 6.2 [18]. Liquid cultures were inoculated with one agar plug (approximately 1 cm × 1 cm) obtained from actively growing PDA cultures per 150 mL of liquid culture medium. The cultures were incubated at 25 °C for 8 days under agitation at 170 rpm to promote mycelial growth. Under these conditions, Tinajero et al. [18] reported a mycelial concentration of approximately 21 g L^−1^ in the liquid culture. This value was not experimentally determined in the present study. Before being used as inoculum, the liquid culture was homogenized using a BLACK+DECKER HB2400B hand blender (Towson, ML, USA) to obtain a more uniform distribution of the fungal inoculum, as fungal growth tends to form aggregates. This procedure was applied consistently to all experimental treatments.

### 2.5. Experimental Design

A reduced central composite design (CCD)-based experimental approach was used to explore the effects of the selected variables in the SSF conditions. This approach minimized the number of experimental runs while still allowing the evaluation of linear, quadratic, and interaction effects of the variables. The independent variables were the inoculum load (x_2_) and the substrate composition (x_1_), expressed as the percentage of sawdust in the solid medium. In this case, the inoculum load (x_2_) was defined as a volumetric ratio (*v*/*w*), referring to the volume of homogenized liquid culture added per mass of solid substrate. Given that each experimental unit had 100 g of solids, the inoculum load numerically corresponds to the volume of inoculum (mL) added.

The solid substrate was formulated as a binary mixture of sawdust and brewer’s spent grain (BSG), where both components are complementary and together represent 100% of the solid fraction.

Therefore, the substrate composition is a mixture variable. However, because the proportions of the two components are complementary, the mixture composition can be represented by a single continuous factor in the experimental design, namely the percentage of sawdust. The percentage of BSG was consequently determined by the relationship:

*BSG* (%) = 100 − *Sawdust* (%)

This representation avoids redundancy between the two component proportions and allows the effect of substrate composition to be evaluated using a single continuous factor.

Although sawdust and BSG are interdependent components of the mixture, changes in the percentage of sawdust necessarily result in complementary changes in the percentage of BSG. Therefore, the effect of the substrate composition was interpreted based on the sawdust proportion while considering the corresponding BSG proportion.

The treatments were defined according to the experimental matrix shown in Table 1. Each experimental unit consisted of 100 g of wet substrate placed in an autoclavable polypropylene bag, which facilitated handling and incubation under laboratory-scale solid-state fermentation conditions. This amount was sufficient to support fungal growth while enabling homogeneous mixing and subsequent estimation of the recovered dry fraction. The initial moisture content was set at 53%, following the standardized substrate preparation protocol for mycelium-based biomaterial production, to ensure suitable conditions for fungal colonization and molding. Due to the nature of solid-state fermentation, pH was not directly adjusted.

Additionally, due to the reduced number of experimental runs and the exploratory nature of the design, this approach is not intended for full optimization or predictive modeling, but rather for identifying general trends in the system.

### 2.6. Inoculation and Incubation of Experimental Treatments

The experimental units were inoculated with previously homogenized liquid culture to promote uniform inoculum distribution throughout the substrate. The inoculum load reported in Table 1 is expressed as a percentage (*v*/*w*), defined as the volume of liquid culture added (mL) per 100 g of substrate. Because the substrate mass was fixed at 100 g per experimental unit, the numerical value of the inoculum load corresponded to the volume of inoculum added in milliliters. After inoculation, the substrate was manually mixed under sterile conditions to promote uniform colonization.

The treatments were incubated in a Memmert incubator (Memmert GmbH + Co. KG, Schwabach, Germany) at 25 °C for 12 days under static conditions (without agitation). No additional pH control was applied during fermentation. The initial substrate moisture content was adjusted to 53% before inoculation but was not remeasured during or after fermentation. An open container filled with distilled water was placed inside the incubation chamber and replenished periodically to maintain a humid atmosphere and minimize substrate moisture loss. All substrate preparation and inoculation procedures were performed under aseptic conditions. No visible contamination was observed in any experimental unit throughout the incubation period.

### 2.7. Recovered Dry Fraction Estimation

The recovered dry fraction was used as an operational measure to indirectly assess the material recovered from the fermented substrate using a dry-weight method [35]. This fraction may contain residual brewer’s spent grain and sawdust particles that cannot be completely separated from the fungal mycelium. Therefore, it should not be interpreted as a direct or exclusive measurement of fungal biomass.

For each treatment, a 3 g sample of previously dried fermented substrate was suspended in 20 mL of distilled water and agitated to facilitate the release of recoverable material. The suspension was then filtered through a sieve, and the retained fraction was transferred to pre-dried and pre-weighed aluminum containers. The recovered material was dried for 24 h and weighed.

The recovered dry fraction was expressed in g L^−1^ by dividing the mass of recovered dry material (g) by the volume of the analytical suspension (0.020 L). Thus, this unit represents the mass of recovered dry material per unit volume of the analytical suspension and does not represent the volumetric concentration of fungal biomass in the original solid-state fermentation system. This operational measurement was used to compare experimental treatments and should not be interpreted as a direct quantification of pure fungal biomass. Measurements were performed in triplicate for each experimental treatment, and the reported values correspond to the mean of the three replicates.

### 2.8. Estimation of Kinetic Parameters

Based on the response surface model, an additional cultivation was performed using 40.13% (*w*/*w*) sawdust, 59.87% (*w*/*w*) BSG, and an inoculum load of 2.69% (*v*/*w*). The solid-state fermentation was conducted for 15 days, and the recovered dry fraction was measured every three days to characterize its temporal evolution.

The apparent specific growth rate (µ) over the observed apparent exponential growth interval (days 3–6) was estimated using Equation (4) [36], where X_1_ and X_2_ represent the recovered dry fraction concentrations (g L^−1^), as operationally defined in Section 2.7, at times t_1_ and t_2_, respectively. The corresponding doubling time (*td*) was calculated using Equation (5).

(4) $$\mu =\frac{\ln\;x_{f}-\ln\;x_{o}\;}{t_{f}-t_{o}}$$

(5) $$\mathrm{td}=\frac{\ln\;\left(2\right)\;}{\mu_{\max}}$$

### 2.9. Biomaterial Production and Mechanical Testing

The treatment that showed the best performance in the experimental design was selected for biomaterial production (40.13% (*w*/*w*) sawdust, 59.87% (*w*/*w*) BSG, and 2.69% (*v*/*w*) inoculum). All specimens were obtained from a single cultivation carried out under these conditions; therefore, the three specimens described below constitute technical replicates from the same production batch and not independent biological or production replicates.

The cylindrical specimens were prepared using molds with dimensions based on the geometrical requirements of ASTM D1621 [37], namely a minimum cross-sectional area of 25.8 cm^2^ and a minimum height of 2.54 cm. The colonized substrate was transferred into molds and manually compacted to obtain the desired cylindrical shape. The specimens were then incubated in the molds at 25 °C for 12 days to allow complete mycelial growth. After this period, the specimens were removed from the molds and dried at 100 °C until a weight reduction of approximately 60–75% was achieved [32].

Before testing, the loading faces of the dried specimens were sanded flat and parallel; because of this operation, the final height of two of the three specimens (24.05 and 24.25 mm) was slightly below the 2.54 cm reference height, while the third specimen measured 26.50 mm. The specimens were stored in sealed polyethylene bags for 34 to 38 days between production and testing and were tested at ambient laboratory conditions, without explicit real-time tracking of temperature and relative humidity.

Compression tests were carried out at the Department of Materials and Models Testing, Faculty of Engineering and Applied Sciences, Universidad Central del Ecuador (Quito, Ecuador), following the Ecuadorian standard NTE INEN 1573 [38]. for the compressive strength of cylindrical specimens (test report No. 16633), using a Matest concrete compression testing machine (Matest S.p.A., Treviolo, Italy) with a 1500 kN capacity, at a loading rate of 0.25 ± 0.05 MPa/s. Height and diameter were measured by the laboratory as average values prior to testing. The load was applied along the axis of the cylindrical specimens, which corresponds to the molding direction. Failure occurred by crushing in all three specimens, and the maximum load sustained by each specimen was recorded. Compressive strength was calculated as the maximum load divided by the initial cross-sectional area, obtained from the average diameter of each specimen.

It should be noted that this criterion differs from that defined in ASTM D1621, which determines compressive strength at the yield point or, in its absence, at 10% deformation; deformation was not recorded during the tests, so the values reported here correspond to the maximum load at crushing failure. Results are therefore reported as the mean ± standard deviation of three technical replicates and are intended as an indicative estimate of the compressive behavior of the material.

## 3. Results

### 3.1. Raw Materials Characterization

The ash content of the sawdust and brewer’s spent grain (BSG) samples was determined using Equation (1), yielding values of 0.93% and 6.08%, respectively. Nitrogen content was calculated using the Kjeldahl method and Equation (2), resulting in values of 0.25% for sawdust and 1.58% for BSG. Furthermore, the estimated protein content was 1.56% for sawdust and 9.88% for BSG (Equation (3)).

As expected, BSG exhibited considerably higher ash, nitrogen, and protein contents than sawdust, reflecting its greater nutritional value and suggesting that it may provide a richer substrate for fungal growth during solid-state fermentation.

### 3.2. Estimate Recovered Dry Fraction and Experimental Design

The recovered dry fraction values obtained for each treatment after 12 days of growth are presented in Table 1. Values are reported as the mean of three independent replicates. A clear variation in recovered dry fraction can be observed depending on the conditions evaluated in the CCD. Treatments 2, 9, and 10 showed the highest recovered dry fraction values, reaching 3.208, 3.155, and 3.138 g/L, respectively. In contrast, Treatments 3 and 6 presented the lowest recovered dry fraction values (1.517 and 1.703 g/L, respectively). These differences indicate that substrate composition and inoculum load were associated with variation in the recovered dry fraction under the evaluated conditions. However, because the recovered fraction may contain residual BSG and sawdust particles, these values should not be interpreted as direct measurements of mycelial growth or pure fungal biomass.

The experimental data were fitted to a second-order polynomial model using R software (version 4.3.3; R Foundation for Statistical Computing, Vienna, Austria), accessed through the RStudio (Version 2026.07.1+147) environment on Posit Cloud (https://posit.co, Posit Software, PBC, Boston, MA, USA) to evaluate the effects of substrate composition and inoculum load on the recovered dry fraction. The statistical significance of the regression coefficients was assessed through ANOVA and *t*-tests of the fitted model terms.

The results showed that the substrate composition factor (x_1_) was statistically significant at both the linear and quadratic levels (*p* < 0.05), indicating that the relative proportions of sawdust and BSG were significantly associated with variation in the recovered dry fraction. In contrast, the inoculum load factor (x_2_) did not show a statistically significant effect within the evaluated range.

The fitted model explained 83.5% of the experimental variability in recovered dry fraction. This value indicates the proportion of variability explained by the fitted model but should be interpreted considering the exploratory nature of the reduced CCD and its associated limitations. Because the reduced central composite design employed in this study included a limited number of experimental points and replicates, formal residual analysis and lack-of-fit assessment could not be robustly performed. Therefore, the fitted polynomial model should be interpreted as an exploratory approximation of the relationship between the evaluated factors and the response variable.

(6) $$Y=3.145644-0.415474x_{1}+0.065637x_{2}-0.093500x_{1}x_{2}-0.541087x_{1}^{2}-0.407039x_{2}^{2}$$

where *Y* represents the predicted recovered dry fraction, *x*_1_ corresponds to the substrate composition factor (sawdust–BSG ratio), and *x*_2_ represents inoculum load.

Based on the fitted model, the conditions associated with the highest recovered dry fraction for *Ganoderma lucidum* growth under SSF were 40.13% (*w*/*w*) sawdust, 59.87% (*w*/*w*) BSG, and 2.69% (*v*/*w*) inoculum load.

To better understand the relative effects of the evaluated variables on the response, a Pareto chart was constructed (Figure 1). This chart is based on standardized effects (t-values), which makes it easier to compare each factor with the critical t-value at a 95% confidence level. As shown, the substrate composition (x_1_) and its quadratic term have the strongest effect on recovered dry fraction, clearly exceeding the significance threshold. On the other hand, the inoculum load and the interaction between variables show a much smaller contribution, which is consistent with the statistical results presented in Table 2.

Visual inspection revealed differences in substrate colonization among treatments. Treatments with higher recovered dry fraction generally exhibited denser and more homogeneous mycelial coverage than treatments with lower values. As shown in Figure 2 and Figure 3, treatments 2, 8 and 10 exhibited a noticeable liquid-like material surrounding the colonized substrate, which may be associated with extracellular polysaccharides (EPS) production or other soluble metabolites. These observations provide qualitative information on colonization patterns but do not independently confirm the recovered dry fraction measurements, as no image analysis or specific EPS quantification was performed.

Figure 4 illustrates the response surface generated from the fitted polynomial model.

The fitted response showed higher recovered dry fraction values toward the central region of the experimental domain, corresponding to intermediate sawdust proportions and inoculum loads. The concave shape of the fitted surface suggests a nonlinear relationship between the evaluated factors and the response within the experimental range. In particular, substrate composition showed a stronger influence on the recovered dry fraction than inoculum load, consistent with the statistical results presented in Table 2.

The region of higher fitted response was associated with a sawdust proportion of approximately 40% and a BSG proportion of approximately 60%, together with an inoculum load of approximately 2.7% (*v*/*w*). These conditions were identified from the fitted response surface and subsequently selected for a separate cultivation to further examine the growth kinetics and to produce the mycelium-based biomaterial. Because the reduced CCD was exploratory and no independent validation experiment was performed, these conditions should not be interpreted as an experimentally validated optimum.

### 3.3. Growth Kinetics and Estimation of Apparent Kinetic Parameters

To further evaluate the selected SSF conditions and characterize the growth kinetics of *Ganoderma lucidum*, a new cultivation was performed using the conditions estimated by the response surface model (40.13% (*w*/*w*) sawdust, 59.87% (*w*/*w*) BSG, and 2.69% (*v*/*w*) inoculum). The recovered dry fraction was monitored every three days for 15 days, and the resulting growth curve is shown in Figure 5.

Based on Equation (4), the apparent specific growth rate (µ) was estimated using the recovered dry fraction values recorded between days 3 and 6, corresponding to the observed apparent exponential growth interval.

The recovered dry fraction profile showed a distinct growth pattern, characterized by a rapid increase between days 3 and 6, followed by an approximately stationary phase between days 6 and 12 and a subsequent decrease. The apparent specific growth rate estimated over the observed interval (days 3–6) was 0.6346 day^−1^, corresponding to a doubling time of 1.092 days. Because these estimates were derived from changes in the recovered dry fraction rather than from a direct measurement of pure fungal biomass, they should be interpreted as approximate, interval-specific estimates. Furthermore, only two observations (days 3 and 6) were available within this interval, limiting the robustness of the kinetic estimates.

### 3.4. Biomaterial Production

The biomaterials were produced using a single cultivation under the conditions identified from the fitted response surface (40.13% (*w*/*w*) sawdust, 59.87% (*w*/*w*) BSG, and 2.69% (*v*/*w*) inoculum load). Three cylindrical specimens were subsequently obtained from this cultivation using molds with a minimum area of 25.8 cm^2^ and a minimum height of 2.54 cm. After drying, the specimens showed a weight reduction of approximately 65%, which falls within the target range (60–75%) established for biomaterial stabilization (Table 3).

The three biomaterial specimens are shown in Figure 6. Their white appearance was consistent with visible fungal mycelial growth on the substrate.

In Table 4, the dimensions of the three specimens, along with their respective weight, volume, density, and compressive strength, can be observed. During mechanical testing to determine the compressive strength, the specimens had to be filed so that they had a flat edge without roughness. For this reason, some weight was lost in biomaterials.

The three biomaterial samples presented densities ranging from 0.24 to 0.27 g cm^−3^ with an average of 0.257 g cm^−3^, a standard deviation of 0.015 and a coefficient of variation of 5.95%. Furthermore, the biomaterial samples exhibited a compressive strength between 550 and 700 kPa, with a mean of 613.33 kPa, a standard deviation of 77.67 and a coefficient of variation of 12.66%. Specimen 1 presented the highest compressive strength (700 kPa), whereas specimen 3 showed the greatest density (0.27 g cm^−3^).

## 4. Discussion

### 4.1. Raw Material Composition

One limitation of the present study is that the characterization of the raw materials was limited to ash, nitrogen, and protein contents, as these parameters could be determined using the analytical methods available for this study and were considered sufficient to evaluate the nutritional characteristics of the substrates for fungal cultivation. A more comprehensive lignocellulosic characterization, including cellulose, hemicellulose, lignin, and starch contents, would provide additional information on substrate composition and could help explain fungal colonization and biomaterial performance. Therefore, future studies should incorporate these analyses to achieve a more complete physicochemical characterization of the selected residues.

According to Serret et al. [39], sawdust in general has an ash content ranging from 0.5 to 2%. This percentage may vary depending on the type of tree from which the sawdust originates. Cedar is one of the main timber trees in Ecuador [40]. Boadu et al. [41] reported an ash content of 2.26% in mixed sawdust from three tropical woods. Although these values differ from the result obtained in the present study, it falls within the general range reported by Serret et al. [39]. This variation in ash content can be attributed to differences in the type of wood used, since sawdust composition depends strongly on the species of origin. In this context, the ash content of 0.93% obtained in this study is particularly relevant, as no previous reports were found for sawdust derived from *Pigue*. Therefore, this result may contribute to future studies, especially considering that this species is native to the Ecuadorian Amazon region.

Regarding the ash content of BSG, previous studies have reported values ranging from 0.52% to 4.6%, depending on beer type, raw materials, and the malting process [27,42,43]. The BSG used in this study showed a higher ash content (6.08%), which may be attributed to the modified low-glucan malts and specific brewing process employed by Andes Brewing Co., as well as differences in grain composition. The higher mineral content of this BSG may have contributed to fungal growth and the greater recovered dry fraction observed.

The nitrogen content of the sawdust (0.25%) was also higher than values previously reported for pine sawdust (0.14–0.18%) [39,44]. This difference is likely associated with the wood species used, as nitrogen content varies according to its botanical origin, and may have increased nutrient availability for fungal growth.

Previous studies have reported nitrogen contents ranging from approximately 3 to 5% in brewer’s spent grain [45,46,47]. The value obtained in this study (1.58%) was lower, probably reflecting differences in grain composition and brewing conditions.

Like the ash percentage, this difference may be attributed to the quality of the grain and the brewing process employed by the brewery from which the BSG is collected. Although the nitrogen content was lower than the values reported in other studies, the results indicate that the substrate formulation supported colonization.

### 4.2. Exploratory Response Surface Analysis

Response Surface Methodology (RSM) integrates regression analysis and experimental design to evaluate the effects of independent variables on a response variable and to characterize relationships among factors, including interaction and quadratic effects [48,49]. Unlike the one-factor-at-a-time (OFAT) approach, RSM allows multiple variables to be evaluated simultaneously and can provide a mathematical description of the response within the experimental domain. In the present study, RSM was used as an exploratory approach to characterize the effects of substrate composition and inoculum load on the recovered dry fraction under the conditions evaluated.

Among the different RSM designs, the Central Composite Design (CCD) provides an efficient framework for evaluating linear, interaction, and quadratic effects and for generating response surfaces [50]. Therefore, a reduced CCD was used in this study to explore the relationship between substrate composition, inoculum load, and the recovered dry fraction during *G. lucidum* SSF. Given the reduced number of experimental runs and the absence of independent validation experiments, the resulting response surface should be considered an exploratory representation of the observed relationships rather than a validated predictive or optimization model.

The fitted quadratic model indicated that substrate composition was the main factor associated with variation in the recovered dry fraction, whereas inoculum load had a comparatively smaller influence within the evaluated experimental domain. The concave shape of the fitted response surface suggests a nonlinear relationship between the evaluated factors and the response [51]. Within the experimental domain, the fitted response was higher toward the central region of the surface, where intermediate proportions of sawdust and BSG were combined with an intermediate inoculum load. This pattern is consistent with the statistical significance of the linear and quadratic terms associated with substrate composition.

The interpretation of the fitted model should be considered in the context of solid-state fermentation systems, which are inherently complex.

The fitted model explained 83.5% of the experimental variability, which is considered satisfactory for biological solid-state fermentation systems. Kinetic modelling in SSF is challenging due to the difficulties involved in accurately measuring growth parameters and substrate consumption [52]. In addition, the heterogeneous nature of the substrate contributes to variability in the system, as these materials are structurally and nutritionally complex [52]. From a modelling perspective, it is important to note that model adequacy should not be evaluated based solely on the R^2^ value, but rather through statistical tools such as ANOVA, which provide a more reliable assessment of model quality [53]. However, it is important to consider that the substrate composition behaves as a mixture variable, which may limit the independent interpretation of factors within the CCD framework. Additionally, the reduced experimental design limits the robustness of the predictive capacity of the model.

### 4.3. Recovered Dry Fraction Quantification and Estimation of Kinetic Parameters

Monitoring fungal growth using gravimetric dry-weight methods in solid-state fermentation is challenging because fungal mycelium cannot be easily separated from the solid substrate, potentially introducing quantification errors [35]. Therefore, this variable represents an operational measure of the recovered dry material rather than a direct quantification of pure fungal biomass. This limitation should be considered when interpreting the response-surface model and the estimated kinetic parameters. Complementary methods, such as respiration-rate measurements, ATP or enzymatic activity assays, immunological detection, and analysis of cell-wall components such as chitin, glucosamine, or ergosterol, could provide more specific estimates of fungal biomass in future studies [54].

Most studies on the growth kinetics of *G. lucidum* have been conducted using liquid culture media. Tinajero reported a maximum specific growth rate of 0.410 day^−1^ in a medium containing starch and yeast extract [18]. Heydarian et al. [55] reported a value of 0.276 day^−1^ in a liquid medium containing glucose, peptone, and yeast extract, whereas a value of 0.230 day^−1^ was reported in a medium containing lactose, peptone, and yeast extract [56].

Fewer studies have evaluated the growth kinetics of *G. lucidum* under solid-state conditions. Angelova et al. [57] reported specific growth rates of 0.267 day^−1^ on wheat straw and 0.513 day^−1^ on pine sawdust. In the present study, the apparent specific growth rate estimated from the recovered dry fraction during the observed interval between days 3 and 6 was 0.6346 day^−1^, which is numerically higher than the values reported in these previous studies. However, this comparison should be interpreted cautiously because the present estimate was derived from changes in the recovered dry fraction, which may include residual substrate particles, and differences in measurement methods and experimental conditions may affect the reported values. The substrate combination used in this study, consisting of sawdust and BSG, may also have contributed to the observed response by combining a lignocellulosic structure with a nutrient-containing component.

The observed growth profile is consistent with the characteristics of SSF, in which microorganisms grow on moist solid substrates and are affected by the physical structure, moisture availability, nutrient distribution, and accessibility of the substrate. SSF can be advantageous for the valorization of agricultural and agro-industrial residues because it can support fungal growth while requiring relatively low water inputs and providing a structured environment for mycelial development [32]. In the present study, the use of sawdust and BSG provided a combination of physical support and nutrients that supported the development of *G. lucidum*.

Several agro-industrial and lignocellulosic residues have been investigated as substrates for the production of mycelium-based biomaterials, including wheat straw, rice straw, hemp hurds, sawdust, coffee grounds, banana leaves, peach palm leaves, and brewer’s spent grain. Previous studies have demonstrated that substrate composition strongly influences fungal colonization, density, and the mechanical performance of the resulting biomaterials. More recently, brewer’s spent grain has also been combined with other agricultural residues for the production of *Pleurotus sajor-caju* mycelium-based composites, highlighting its potential as a nutrient-rich substrate within circular bioeconomy strategies [13]. In the present study, the combination of brewer’s spent grain and sawdust was selected to integrate the nutritional advantages of BSG with the structural support provided by sawdust, suggesting that these abundant agro-industrial residues can be successfully valorized for the production of *Ganoderma lucidum*-based biomaterials with promising mechanical properties under the conditions evaluated in this study.

The recovered dry fraction values obtained across treatments highlight the association between substrate composition and the measured response. Sawdust provides a lignocellulosic structure that may support hyphal attachment and development, whereas BSG generally provides a greater supply of nutrients [26]. Treatment 2, which contained 75% BSG, showed the highest recovered dry fraction, while Treatments 9 and 10, which contained a balanced substrate composition, also showed relatively high values. Conversely, Treatments 3, 4, and 6, which contained higher proportions of sawdust, showed lower recovered dry fraction values. These observations suggest that substrate composition influenced the amount of dry material recovered after fermentation. However, because the recovered fraction may contain residual substrate particles, the differences cannot be attributed exclusively to differences in fungal biomass.

Additionally, increasing the inoculum load did not necessarily result in a higher recovered dry fraction. For example, in Treatment 8, despite having a balanced substrate composition, the higher inoculum load did not result in the highest recovered dry fraction. This may be explained by the fact that a higher initial recovered dry fraction can accelerate nutrient consumption, leading to an earlier depletion of available nutrients and limiting further growth. Therefore, higher inoculum loads did not necessarily result in higher recovered dry fraction values after the 12-day fermentation period.

The liquid-like material observed in Treatment 2, 8 and 10 may be related to the production and accumulation of extracellular polysaccharides (exopolysaccharides) during mycelial growth. Fungal polysaccharides can occur as homopolysaccharides, heteropolysaccharides, and glycoproteins, and can be found both within fungal cells and in the surrounding environment [58]. Exopolysaccharides are released by fungi into the surrounding medium and have been associated with protective functions. The production of exopolysaccharides can be influenced by factors related to culture conditions [59,60]. Therefore, the presence of the liquid-like material in Treatment 8 could be related to differences in fungal colonization and metabolic activity among treatments. However, since exopolysaccharides production was not directly quantified or chemically characterized in this study, this interpretation should be considered a possible explanation rather than a confirmed result.

The recovered dry fraction profile obtained under the model-identified conditions provides information relevant to defining the cultivation period for subsequent biomaterial production. The recovered dry fraction increased during the initial phase and reached approximately 7 g/L by day 6, remained relatively stable through day 12, and decreased by day 15. This pattern describes changes in the recovered dry material over time but cannot be interpreted exclusively as changes in fungal biomass because residual substrate particles may contribute to the measured fraction. Nevertheless, the observed profile provides a preliminary basis for selecting sampling times and cultivation periods for further investigation.

Therefore, identifying the apparent growth interval and subsequent stabilization of recovered dry fraction is relevant when defining the cultivation period for mycelium-based biomaterial production.

Finally, the present results demonstrate the potential of SSF using locally available residues such as sawdust and BSG for *G. lucidum* cultivation. Nevertheless, additional experiments with a higher sampling frequency during the early growth phase would be required to obtain a statistically robust estimation of µmax and to more precisely characterize the growth kinetics of the fungus under these conditions.

### 4.4. Density and Compressive Properties of Mycelium-Based Biomaterials

The mechanical results reported here were obtained from three technical replicates produced in a single batch and therefore provide an indicative range of compressive strength for this substrate formulation rather than a statistically robust mechanical characterization; between-batch variability remains to be determined. Additionally, compressive strength values reflect the maximum load sustained at crushing, as deformation was not recorded during testing. Consequently, strain-based criteria of ASTM D1621 (e.g., yield point or stress at 10% deformation) could not be evaluated, and comparisons with literature reporting ASTM D1621 values should be interpreted with caution.

In the present analysis, biomaterial samples can be classified as Mycelium-Based Foams (MBF), as they originate through the colonization of a substrate by fungi, resulting in a structure with high porosity and lightweight rigidity. According to Girometa et al. [60], mycelium-based biomaterials are divided into two categories: foams and sandwich-type compounds. MBF are low-density materials formed when fungi colonize a lignocellulosic substrate, creating an anisotropic fibrous structure composed of fungal mycelium and residual substrate particles. Although it does not exactly match the definition of a “solid foam,” it is classified as such due to its high porosity and lightness.

The competitiveness of MBF is significantly influenced by its low density. This low density is advantageous for packaging applications because it reduces the overall material weigh, which is advantageous for packaging applications. The decrease in MBF final density is closely related to porosity and the progressive replacement of the solid substrate by the fungal mycelial network, leading to a decrease in the density of the composite material [61].

These comparisons should be interpreted with caution, as the values compiled in Table 5 were obtained using different testing standards and criteria. Where compressive strength was determined according to ASTM D1621, it corresponds to the stress at the yield point or at 10% deformation, whereas the values reported here correspond to the maximum load sustained at crushing failure (Section 2.9). Because the maximum load occurs later in the compressive response than the yield point, this criterion tends to produce higher values; the differences shown in Table 5 therefore reflect, in part, differences in measurement criteria and specimen geometry rather than material performance alone.

In this study, the mycelium-based specimens produced in this study exhibited densities ranging from 0.24 to 0.27 g/cm^3^ (Table 5). These values fall within the range reported for mycelium composites produced from different lignocellulosic substrates (0.10–0.39 g/cm^3^) [8] and are comparable to those obtained for *Ganoderma lucidum* grown on beech sawdust (0.215 g/cm^3^) [62]. In contrast, Islam et al. [63] reported a substantially lower density (0.05 g/cm^3^) which was approximately 5.2-fold lower than the value obtained in the present study; however, their material consisted exclusively of compressed mycelium rather than a composite containing lignocellulosic residues, making direct comparisons difficult. Although the density of the developed biomaterials was lower than that of extrusion-grade polystyrene (1.05 g/cm^3^) [69], their lightweight nature highlights their potential for sustainable packaging applications. The density of polystyrene is approximately 3.89 times higher than the density of the mycomaterial obtained in this research.

As shown in Table 5, the compressive strength of the biomaterials ranged from 550 to 700 kPa. These values are within the upper range reported for mycelium-based composites produced using different fungal species and substrates [22,64,65] and are considerably higher than those reported for biomaterials produced from *Ganoderma lucidum* or *Pleurotus* spp. grown on wheat, barley, cotton, or rice husk substrates [17,66,67,68]. The relatively high compressive strength obtained in this study is likely associated with the balanced combination of BSG and sawdust. While BSG provides nutrients that promote extensive mycelial colonization, sawdust contributes structural support and a lignocellulosic framework that favors the formation of a compact composite [29]. The interaction between these components likely improved the mechanical integrity of the resulting biomaterial. Compared with the study by Holt et al. [66], which used processed cotton biomass as a substrate, the compressive strength reported here was approximately nine times higher than their reported value of 72 kPa. A study on *Ganoderma lucidum* indicates that biomaterials made from wheat and barley have a minimum compression strength of 44.17 kPa and a maximum of 131.21 kPa [17], both of which are lower than the values obtained in this investigation.

Overall, the compressive strength values obtained in this study (550–700 kPa) were higher than those reported in previous studies involving *Pleurotus ostreatus* and *Ganoderma lucidum*. Although these values fall within the range reported by Ziegler et al. [64], differences in fungal species, substrate composition, and testing conditions should be considered when comparing results across studies. Furthermore, the use of the fungal species in the present study should be considered when interpreting these findings.

Regarding conventional expanded polystyrene (EPS), reported compressive strength values are approximately 89 kPa for EPS13 (density of 13.5 kg/m^3^) and 191 kPa for EPS28 (density of 28 kg/m^3^) [70]. Higher values, ranging from 690 to 4060 kPa, have been reported for EPS-based composite materials, depending on their composition and structural configuration [71,72]. A compressive strength of approximately 690 kPa has also been reported for EPS used in packaging applications [21]. Thus, the values obtained for the mycomaterials fall within the range reported for certain EPS-based materials. However, this comparison is indicative only, as differences in density, composition, and testing conditions limit direct comparisons and do not establish functional equivalence.

These findings suggest that mycomaterials produced using *G. lucidum* and a lignocellulosic substrate composed of brewer’s spent grain (BSG) and sawdust warrant further investigation for potential packaging applications. Nevertheless, compressive strength alone is insufficient to establish their suitability as alternatives to conventional polymeric foams. Further studies are required to evaluate water resistance, dimensional stability, thermal properties, durability, reproducibility across independent production batches and other relevant mechanical characteristics under application-specific conditions.

## 5. Conclusions

This exploratory study evaluated brewer’s spent grain (BSG) and sawdust as substrate components for the solid-state fermentation (SSF) of *Ganoderma lucidum* and the production of mycelium-based biomaterials. Within the evaluated experimental domain, the reduced central composite design and fitted response surface model associated a formulation containing 40.13% (*w*/*w*) sawdust, 59.87% (*w*/*w*) BSG, and an inoculum load of 2.69% (*v*/*w*) with the highest recovered dry fraction. This result should be interpreted as an exploratory model-based estimate rather than as a validated optimum. The apparent specific growth rate estimated from the recovered dry fraction between days 3 and 6 was 0.6346 day^−1^, corresponding to a doubling time of 1.09 days. These kinetic estimates are approximate because they are based on only two observations within the apparent exponential-growth interval.

Substrate composition was the main factor associated with variation in the recovered dry fraction, whereas inoculum load did not show a significant effect within the evaluated experimental domain. However, the recovered dry fraction may contain residual BSG and sawdust particles that cannot be completely separated from the fungal mycelium. Therefore, this response represents an operational measure of recovered dry material rather than a direct quantification of pure fungal biomass, and the observed differences should not be attributed exclusively to changes in fungal growth.

The biomaterials obtained under the model-identified conditions exhibited densities ranging from 0.24 to 0.27 g cm^−3^ and compressive strengths between 550 and 700 kPa. These preliminary measurements provide an initial indication of the physical and mechanical properties of the resulting composites but do not establish their functional equivalence to expanded polystyrene or confirm their suitability for specific packaging applications. Furthermore, the characterization was limited primarily to density and compressive strength, and the three specimens were obtained from a single cultivation batch. Additional independent production batches and broader material characterization are therefore required to assess reproducibility and application-specific performance.

Overall, this study provides an exploratory basis for further investigating the valorization of agro-industrial and wood-processing residues through *G. lucidum* SSF for mycelium-based biomaterial production. Future work should include more specific fungal biomass quantification, additional experimental observations for improved kinetic estimation, independent validation of the model-predicted conditions, and comprehensive material characterization to determine the reproducibility and potential of this approach for larger-scale applications within a circular bioeconomy framework.

## Figures and Tables

**Figure 1:**
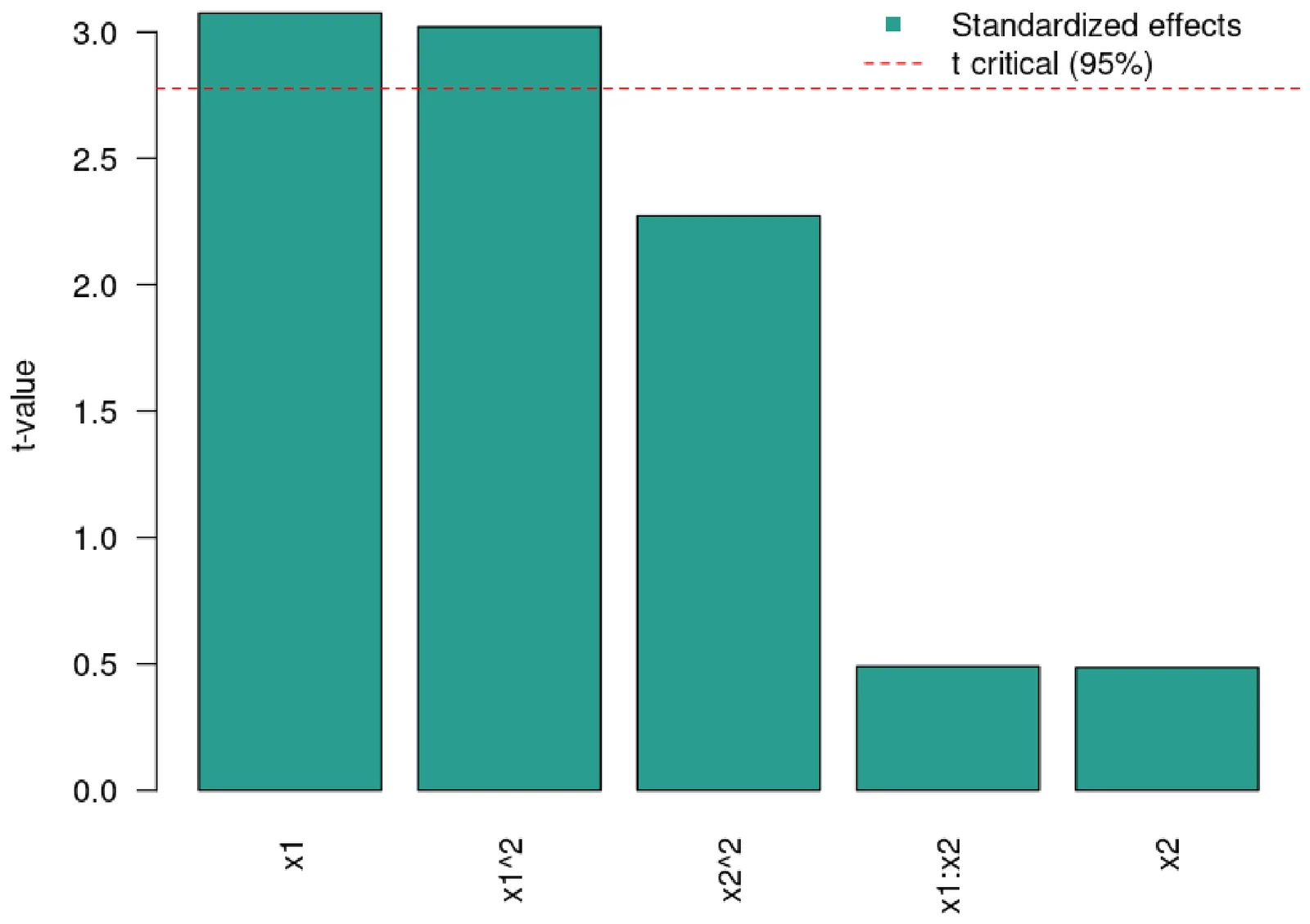
Pareto chart of standardized effects (t-values) on recovered dry fraction. The red dashed line represents the critical t-value of 2.78 at a 95% confidence level.

**Figure 2:**
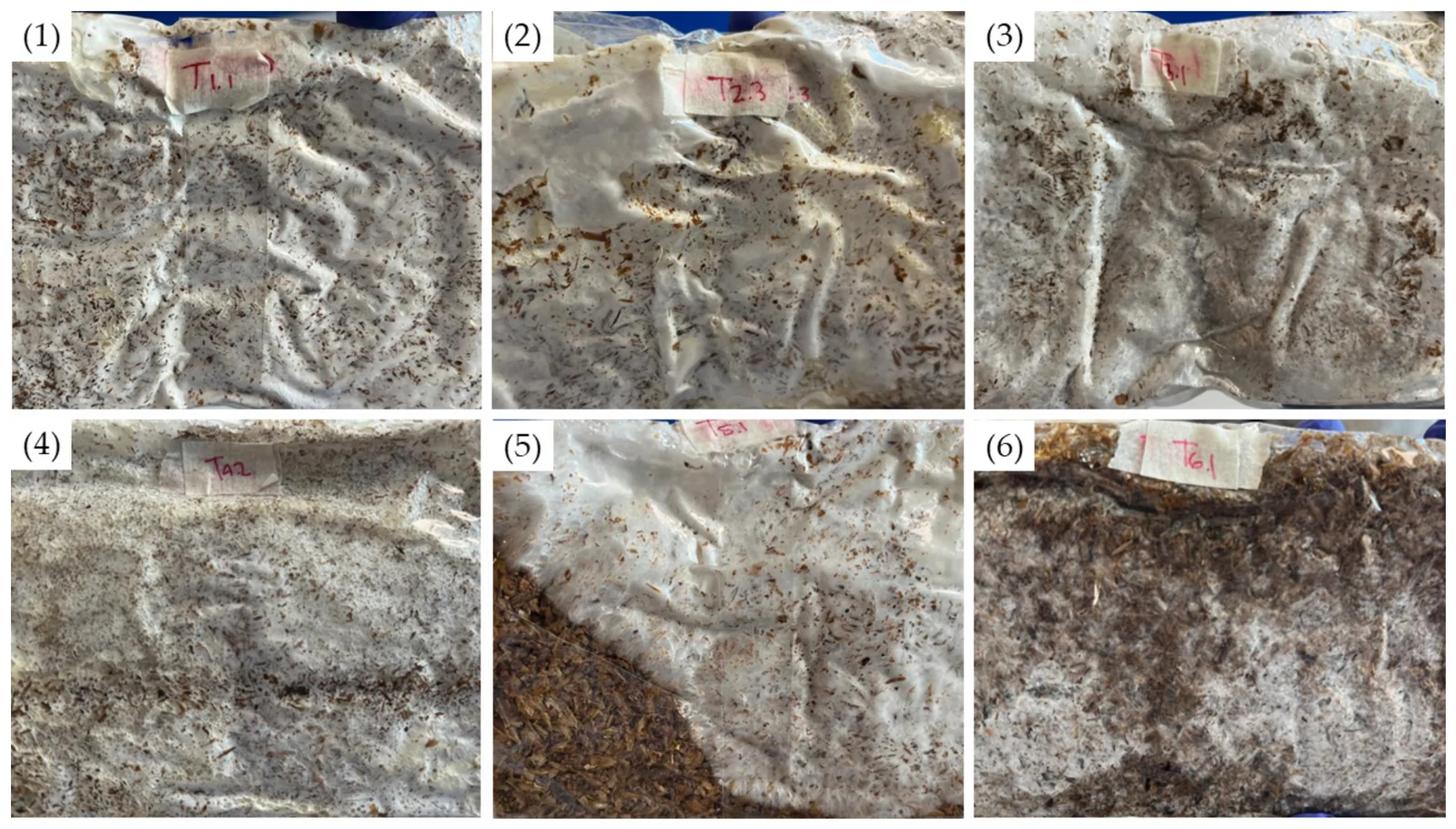
Representative images of mycelial colonization under different substrate formulations and inoculum loads evaluated in the reduced central composite design (CCD): (**1**) Treatment 1: 25% sawdust, 75% brewer’s spent grain (BSG), and 1.00% (*v*/*w*) inoculum; (**2**) Treatment 2: 25% sawdust, 75% BSG, and 4.00% (*v*/*w*) inoculum; (**3**) Treatment 3: 75% sawdust, 25% BSG, and 1.00% (*v*/*w*) inoculum; (**4**) Treatment 4: 75% sawdust, 25% BSG, and 4.00% (*v*/*w*) inoculum; (**5**) Treatment 5: 14.75% sawdust, 85.25% BSG, and 2.50% (*v*/*w*) inoculum; and (**6**) Treatment 6: 85.25% sawdust, 14.75% BSG, and 2.50% (*v*/*w*) inoculum. The images illustrate differences in visible mycelial colonization patterns among the evaluated treatments.

**Figure 3:**
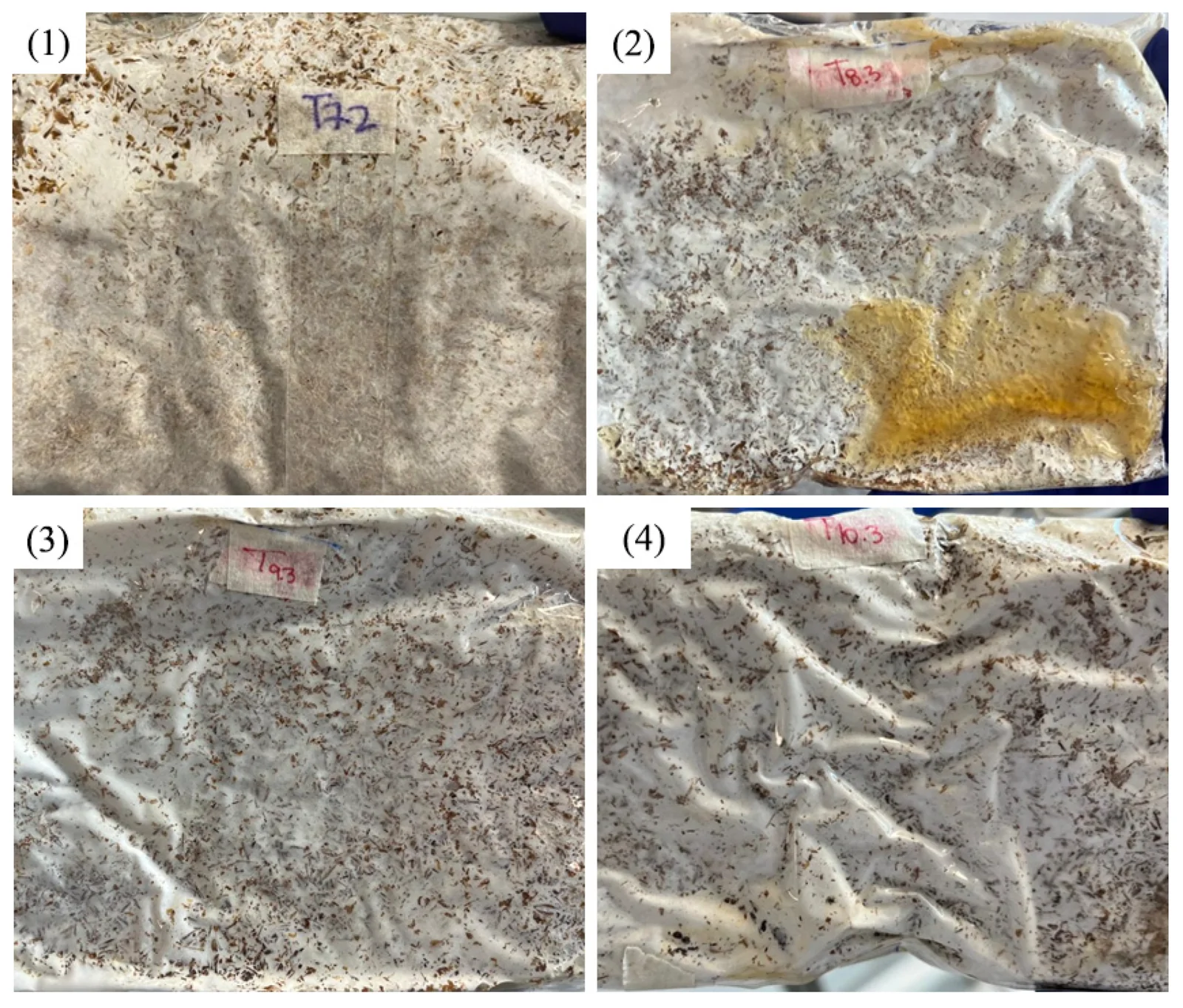
Representative images of mycelial colonization under different substrate formulations and inoculum loads evaluated in the reduced central composite design (CCD): (**1**) Treatment 7: 50% sawdust, 50% brewer’s spent grain (BSG), and 0.385% (*v*/*w*) inoculum; (**2**) Treatment 8: 50% sawdust, 50% BSG, and 4.615% (*v*/*w*) inoculum; (**3**) Treatment 9: 50% sawdust, 50% BSG, and 2.50% (*v*/*w*) inoculum; and (**4**) Treatment 10: 50% sawdust, 50% BSG, and 2.50% (*v*/*w*) inoculum. The images illustrate differences in visible mycelial colonization patterns among the evaluated treatments.

**Figure 4:**
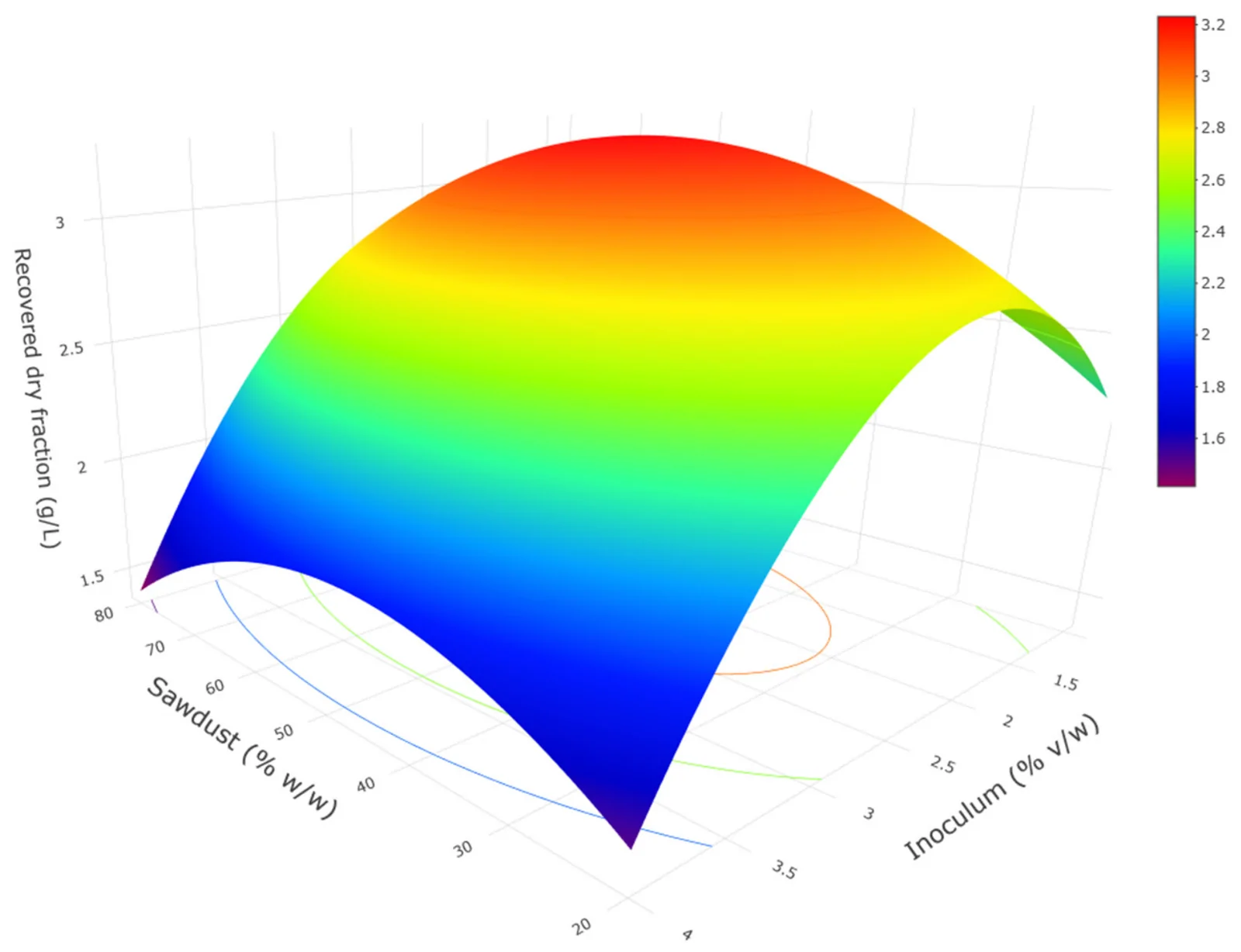
Response surface generated from the reduced central composite design (CCD), showing the fitted recovered dry fraction (g/L) as a function of sawdust proportion and inoculum load. The colored contour lines projected onto the bottom plane represent combinations of sawdust proportion and inoculum load associated with equal predicted values of the recovered dry fraction. The color gradient indicates the magnitude of the predicted response, as shown by the color scale.

**Figure 5:**
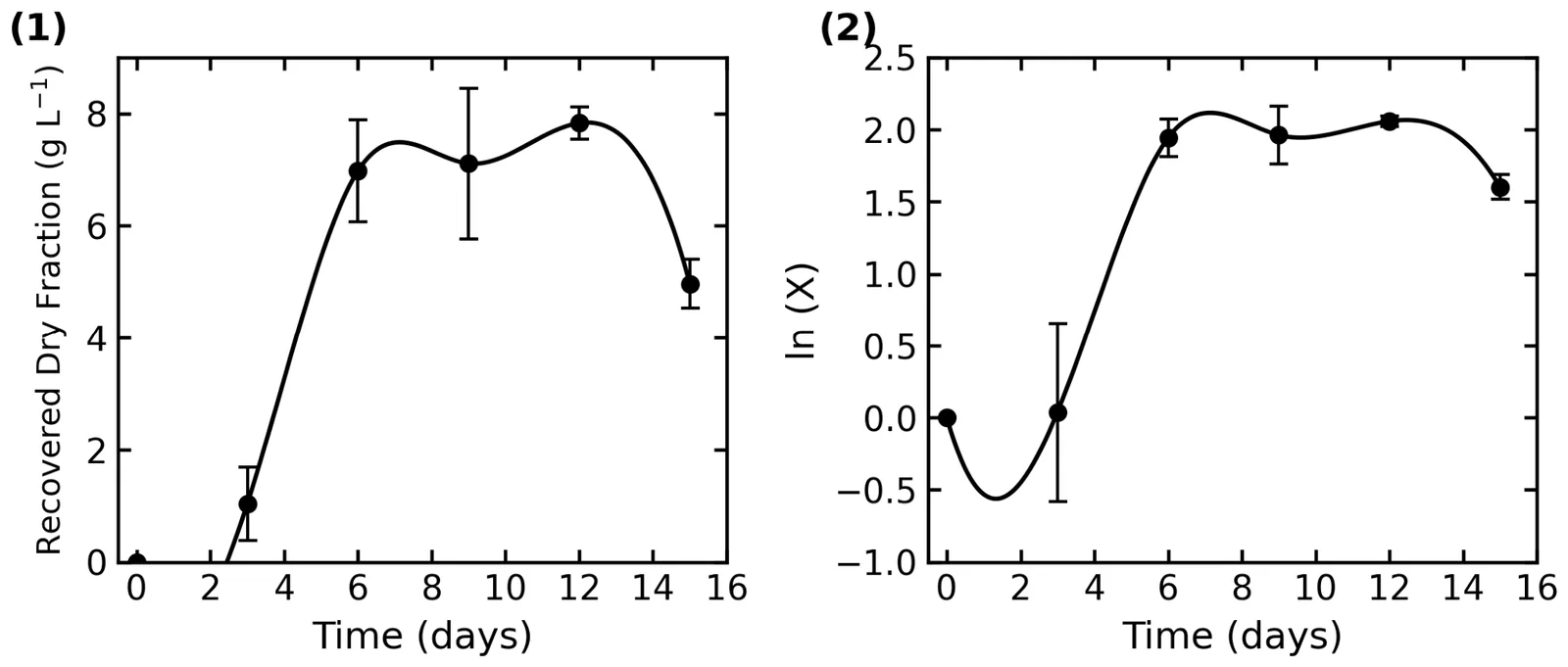
(**1**) Growth kinetics of *Ganoderma lucidum* under the selected solid-state fermentation conditions. (**2**) Logarithmic transformation of recovered dry fraction [ln(X)] as a function of cultivation time.

**Figure 6:**
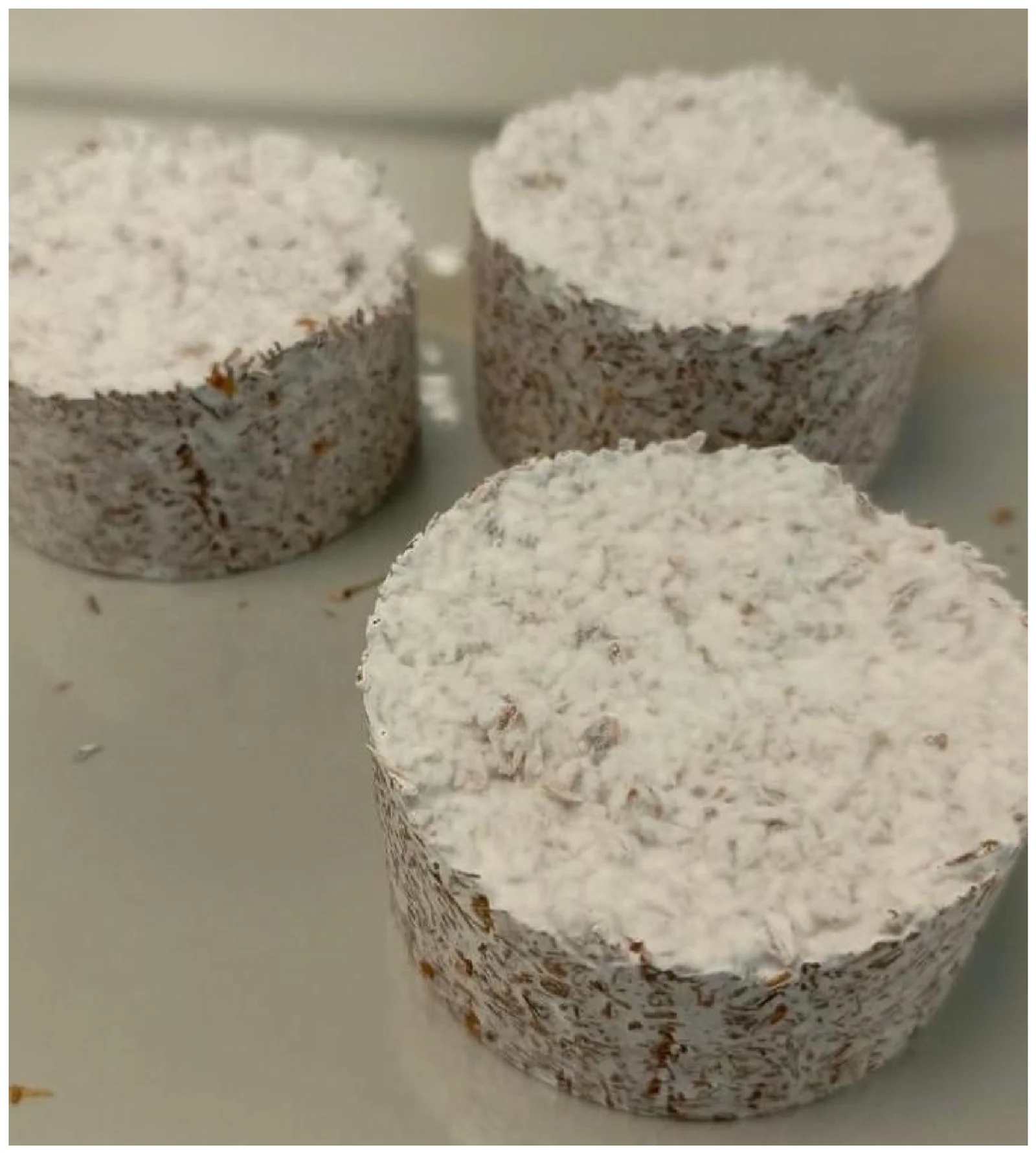
Representative mycelium-based biomaterial specimens produced under the selected SSF conditions.

**Table 1:** Central Composite Design (CCD) matrix and factor levels evaluated in substrate formulation and inoculum load for each treatment.

Treatments	Substrate Formulation			Inoculum Load		
	CCD Matrix (x_1_)	Sawdust % (*w*/*w*)	Brewer’s Spent Grain % (*w*/*w*)	CCD Matrix (x_2_)	Inoculum Load % (*v*/*w*)	Recovered Dry Fraction (g/L)
1	−1	25.00	75.00	−1	1.00	2.571
2	−1	25.00	75.00	1	4.00	3.208
3	1	75.00	25.00	−1	1.00	1.517
4	1	75.00	25.00	1	4.00	1.780
5	−1.41421	14.75	85.25	0	2.50	2.293
6	1.41421	85.25	14.75	0	2.50	1.703
7	0	50.00	50.00	−1.41421	0.385	2.398
8	0	50.00	50.00	1.41421	4.615	2.310
9	0	50.00	50.00	0	2.50	3.155
10	0	50.00	50.00	0	2.50	3.138

Note: (x_1_) and (x_2_) represent the coded values for substrate composition and inoculum load, respectively. Substrate composition is expressed as the percentage of sawdust, with BSG accounting for the remainder. Inoculum load is expressed as the volume of liquid inoculum per mass of substrate (% *v*/*w*). The recovered dry fraction is expressed in g/L of analytical suspension and represents an operational measurement, not a direct quantification of pure fungal biomass.

**Table 2:** Regression coefficients and statistical significance of the fitted quadratic Response Surface Methodology (RSM) model.

Term	Estimate	Std. Error	t Value	*p*-Value	Significance
Intercept	3.145644	0.269797	11.6593	0.0003094	***
x1	−0.415474	0.135102	−3.0753	0.0371024	*
x2	0.065637	0.135102	0.4858	0.6525010	ns
x1:x2	−0.093500	0.190779	−0.4901	0.6497313	ns
x1^2^	−0.541087	0.179140	−3.0205	0.0391451	*
x2^2^	−0.407039	0.179140	−2.2722	0.0855209	.

Note: * = *p* < 0.05; *** = *p* < 0.001; . = *p* < 0.1 and ns = not significant (*p* > 0.05).

**Table 3:** Wet weight of the mycelium specimens and dry weight after being dried in an oven.

Specimens	Wet Weight (g)	Dry Weight (g)	Weight Loss (%)
1	66.99	23.20	65.37
2	71.10	24.86	65.04
3	87.35	31.39	64.06

**Table 4:** Dimensions, weight, volume, density, and compressive strength of the three specimens and compressive strength of the three specimens.

Specimens	Dimensions						
	Diameter (cm)	Height (cm)	Final Weight (g)	Volume (cm^3^)	Density (g/cm^3^)	Maximum Load (kN)	Compressive Strength (kPa)
1	6.66	2.41	20.18	83.96	0.24	2.44	700
2	6.69	2.43	22.01	85.41	0.26	2.06	590
3	6.71	2.65	24.84	93.71	0.27	1.94	550

Note: Compressive strength was calculated as the maximum load divided by the initial cross-sectional area, obtained from the average diameter of each specimen (Section 2.9). Deformation was not recorded during testing; therefore, the reported values correspond to the maximum load sustained at crushing failure and not to the strain-based criterion defined in ASTM D1621.

**Table 5:** Comparison of density and compressive strength of mycelium-based biomaterials reported in the literature.

Study	Fungus	Substrate	Density (g/cm^3^)	Compressive Strength (kPa)
This study	*Ganoderma lucidum*	Sawdust + BSG	0.24–0.27	550–700
Appels et al. [10]	*Pleurotus ostreatus* *Trametes multicolor*	Rapeseed straw + beech sawdust + cotton	0.19–0.39	-
Gaff et al. [62]	*Ganoderma lucidum*	Beech sawdust	0.215	-
Islam et al. [63]	*-*	Compressed mycelium	0.05	-
Ziegler et al. [64]	*Fungal strain combination*	Cotton fluff + Hemp	-	670–1180
Aiduang et al. [22]	*Ganoderma fornicatum* *Ganoderma williamsianum* *Lentinus sajor-caju* *Trametes coccinea* *Schizophyllum commune*	Bamboo Sawdust + Corn Pericarp	0.212–0.282	400–952
Yang et al. [65]	*-*	Wood Pulp + Millet grain + Wheat bran + Natural fiber + Calcium Sulfate	-	350–567
Holt et al. [66]	*Ganoderma* spp.	Compressed Cotton	0.066–0.224	72
Sinailín and Titusunta [17]	*Ganoderma lucidum*	Wheat + Barley	-	44.17–131.21
Garrido [67]	*Pleurotus* spp.	Sawdust + Rice Husk	-	89.63–551
Aparicio et al. [68]	*Pleurotus ostreatus*	Sawdust + Wheat Flour	-	491

Note: Values were compiled from studies using different fungal species, substrates, specimen geometries, and testing standards. Compressive strength determined according to ASTM D1621 corresponds to the yield point or 10% deformation, whereas the values reported in this study correspond to the maximum load at crushing failure; direct comparison should therefore be made with caution.

## Data Availability

All data generated or analyzed during this study are included in this published article. Additional raw data (experimental design and kinetic data) are available from the corresponding author upon reasonable request.

## Abbreviations

The following abbreviations are used in this manuscript:

BSG	Brewer’s Spent Grain
SSF	Solid-State Fermentation
CCD	Central Composite Design
µ	Specific Growth Rate
td	Doubling Time
EPS	Expanded Polystyrene
PDA	Potato Dextrose Agar
ASTM	American Society for Testing and Materials
ANOVA	Analysis of Variance
RSM	Response Surface Methodology
OFAT	One-Factor-At-A-Time
ATP	Adenosine Triphosphate
MBF	Mycelium-based foams
LSF	Liquid-State Fermentation
